# The role of employee psychological stress assessment in reducing human resource turnover in enterprises

**DOI:** 10.3389/fpsyg.2022.1005716

**Published:** 2022-10-10

**Authors:** Cong Liu, Wenqing Miao

**Affiliations:** Department of Business Administration, Weifang University of Science and Technology, Weifang, Shandong, China

**Keywords:** employee psychological stress, condition assessment, human resources, turnover, evaluation model

## Abstract

In this paper, the model is constructed by measuring the psychological stress condition of employees; the psychological stress condition measurement model analyzes and tests the reasons for reducing human resource turnover in enterprises. In this paper, through the research related to the problem of talent loss in enterprises, we found that enterprises of other ownership often use the talent loss early warning model, to measure the possibility of talent loss in enterprises and issue an early warning, which enables enterprises to solve the talent loss crisis in time and minimize the negative impact of talent loss on enterprises. In this paper, we analyze the causes of human resource attrition risk from a theoretical point of view, construct a system of human resource attrition risk indicators for enterprises, and explain the content of each technical indicator and its measurement method. The PLS structural equation of the HR attrition risk evaluation model is established based on the constructed risk index system. In addition, the analysis of the causes of human resources turnover risk in the company also proposes strategies to avoid and prevent the threat. The PLS structural equation evaluation model of HR turnover risk is applied to various situations of human resource management to analyze the case of the comprehensive evaluation of HR turnover risk to guide the practical application of the model. Therefore, the research results of this paper have significant reference value for enterprises to solve similar human resource attrition problems. At the same time, it will provide a reference for enterprises to improve their human resource diagnostic capability and promote the development of human resource management.

## Introduction

With the rise of the information revolution, the rapid development of science and technology, and the rapid promotion of urbanization, human society has transitioned from traditional agricultural and industrial community to modern society, and the advent of the new era has fundamentally changed people’s way of life and work (Orji and Yakubu, 2020). To adapt to social changes, people must spend more time and energy learning new knowledge, skills, and abilities after their careers to adapt to the trend of social development. They are under the pressure of work and life for a long time, and the resulting psychological problems are becoming more and more prominent (Loon et al., 2019). According to the information published by UNESCO, mental illness is the biggest killer of human health in the 21st century. UN experts predict that by the middle of the 21st century, the psychological crisis will bring people pain like a disaster, and this pain cycle is long and impressive. Obsessive–compulsive disorder, depression, anxiety, and other mental illnesses are spreading in society. Modern people are paying the price of mental health for pursuing material life.

In today’s era, with the rapid development of information technology, the globalization of the knowledge economy has become an international macro trend, and competition among enterprises has intensified. It has changed from early price competition to employee competition. How to attract and retain employees and let employees empower the Enterprise to become the basis for enterprises to maintain competitiveness (Pandey, 2020). At present, we are in the transition period of economic and social development due to the change in economic development mode, social structure, and the cognitive change caused by it, which has an important impact on the work attitude and professional behavior of employees (Kurniawaty et al., 2019). The demands for various interests are becoming increasingly diversified, and the expectations for the Enterprise are getting higher and higher. At the same time, to better adapt to the fierce market competition, enterprises continue to improve product quality, service quality, service level, etc., and the requirements for employees are also increasingly high. In the long run, employees are under tremendous pressure, increasing their sense of anxiety and crisis (Ehsan and Ali, 2019). In addition, due to the different perceptions and positions of rights and obligations with enterprises and different ways and perspectives of thinking about problems, it is also easy to cause a decline in trust between the two sides, resulting in a lack of vitality, concentration, lack of enthusiasm, low dedication, and tendency to leave the work of employees, which has a very serious This has a severe impact on the development of the company. The company has a grave personnel turnover and departure rate, resulting in a shortage of personnel, and vacancies in the operation of the Enterprise, which to a certain extent causes a delay in the construction progress of cooperative projects and cannot meet the daily design needs of the project, and has caused adverse impact on the image of the Enterprise. The current situation of personnel turnover is somewhat representative, so it is of theoretical significance and practical significance to study the company’s personnel turnover problem.

Enterprises, especially labor-intensive enterprises, are having more difficulties recruiting and employing workers. Employees for an enterprise are the backbone of development, the Enterprise’s strategic core, and valuable resources. For the garment processing manufacturing industry, in addition to equipment and capital investment to ensure product quality, it is necessary to be competitive in human resources (Erawati et al., 2019). Frontline employees are the source of profits and an essential part of the stable development of the Enterprise. Therefore, the compelling attraction, training, and retention of frontline employees become especially important. In today’s economic downturn environment, there is a shortage of frontline employees in various industries (Yu et al., 2021). The staff turnover rate is increasing yearly, leading to a decline in enterprise productivity and weakening overall competitiveness. The uncontrolled loss of employees will cause instability in the enterprise workforce, leading to a reduction in business efficiency, damage to corporate image, and a series of other problems, seriously limiting the development of enterprises. The company is a processing OEM for small and medium-sized enterprises; the frontline staff turnover problem is severe (Ju et al., 2020). In the past 2 years, the company has lost 220 frontline employees. The loss of frontline employees has led to a workforce shortage and frequent recruitment activities, which has also increased the cost of training new employees, lowered the efficiency of the company’s human resources department, and expanded the company’s employment costs (Khuong and Linh, 2020). Therefore, it is significant for the company to explore the problem of frontline employee turnover. Once the issue of frontline staff turnover is further aggravated without a solution, it will not only cause the company’s productivity to decrease and frequent changes in human resources, leading to chaos in corporate management, but also affect the cooperation with customers and reduce the market competitiveness of the company. In this case, it is a necessary research topic for companies to reduce the turnover of frontline employees and enhance the retention strategy of employees.

## Related works

However, most of the early studies on this issue were conducted from a macro perspective, which included analysis of the different effects of different categories on employee turnover, including specific contemporary causes such as compensation, training, labor market stratification, and social unemployment rates (Meyer et al., 2021). After the emergence of Hawthorne’s experiment, the study of employee turnover provided a new way of thinking about the tendency of employee turnover to organizational behavior. The employee turnover early warning mechanism was initially derived from enterprise early warning theory (Mao et al., 2021). With the continuous development of enterprise crisis management theory research, enterprise crisis early warning management has been gradually refined to different functional levels of enterprises, including financial risk management, marketing risk prediction, and enterprise human resource management crisis research. Enterprise early warning management was first used to monitor and control the financial risks of enterprises, and early warning models have developed from the study of univariate factors to the determination of multi-factor variables for the prediction of financial crises (Yang et al., 2021). The construction of early warning models usually requires analysis, starting from the causes of the problem. In the study of the causes of employee turnover, March and Simon’s study was far-reaching, summarizing the causes of employee turnover into two key reasons: the job satisfaction of the Enterprise’s employees and the ease with which the Enterprise’s employees can flow into other enterprises (Rao et al., 2021). Based on the changing social situation, scholars have explored the traditional turnover models in depth, among which the more typical analytical models are the Price–Mueller model, the March–Simon model, Mobley’s mediation model, and Lee’s unfolding job embeddedness model. These models have proposed two new panels; one is the analysis of the rationality of participants’ outflow from the firm; the other is the analysis of the ease of participants’ flow from the firm.

Employee turnover research has been a hot topic of scholarly research, and experts and scholars have proposed many theories. Most of these theories have been studied by large and medium-sized companies and their employees (Zhu et al., 2021). These large and medium-sized companies have formed a more mature management system in the company system and are at a higher level in the same industry in terms of compensation and benefits, so the proposed measures to alleviate employee turnover tend to be more theoretical and pedantic (Alipour and Harris, 2020). The object of this paper is a representative small and medium-sized manufacturing company and its grassroots employees, whose high turnover rate is a microcosm of many manufacturing companies in the process of transformation and upgrading (Mishra et al., 2019). This paper analyzes the employee turnover of a typical small and medium-sized manufacturing company. It proposes targeted measures to deal with employee turnover, which helps to enrich the management theory of human resources employee turnover and the methods of dealing with employee turnover strategies.

By studying the relationship between mental health burnout and psychological capital among college teachers, Abu Dabous psychological capital partially mediates and moderates the relationship between mental health and burnout (Abu Dabous et al., 2021). The study of psychological money and burnout has a significant negative correlation; psychological health partly mediated the relationship between psychological capital and burnout. Based on this, Chang measured different groups to understand the influencing factors of psychological health (Chang et al., 2019). The results of Zhang study on mobile companies correlated the level of mental health and burnout and coping styles of mobile company employees and that employees’ burnout and coping styles were predictive of their mental health (Zhang et al., 2019). Bao studied the relationship between burnout, coping style, and mental health level of elementary school teachers; burnout directly affects the level of mental health (Bao et al., 2019). An upbeat coping style indirectly affects the group of mental health elementary school teachers who have more significant problems with their mental health level, which a lively coping style can adjust. Mental health is related to various factors, and psychological capital, burnout, and positive coping styles are all related to mental health. Still, most research subjects mainly focus on students, teachers, patients, and box doctors, and not many studies directly on subway employees. It is crucial to conduct a mental health survey for subway employees to explore the factors that cause their mental health. Based on the current state of employees’ mental health, this study examines the effects of burnout and coping styles on them and establishes the mediating role of psychological capital on them (Eknath and Janardhan, 2020). Based on previous studies, this study examines the correlation between employee burnout, coping style, psychological capital level, and mental health. It comprehensively analyzes the relevant factors affecting employees’ mental health in enterprises to provide scientific reference for enterprises to predict the development trend of employees’ mental health, improve work motivation, and perfect employee management methods.

## The role of employee psychological stress assessment in reducing human resource turnover in enterprises

### Employee psychological stress assessment model construction

**Figure 1:**
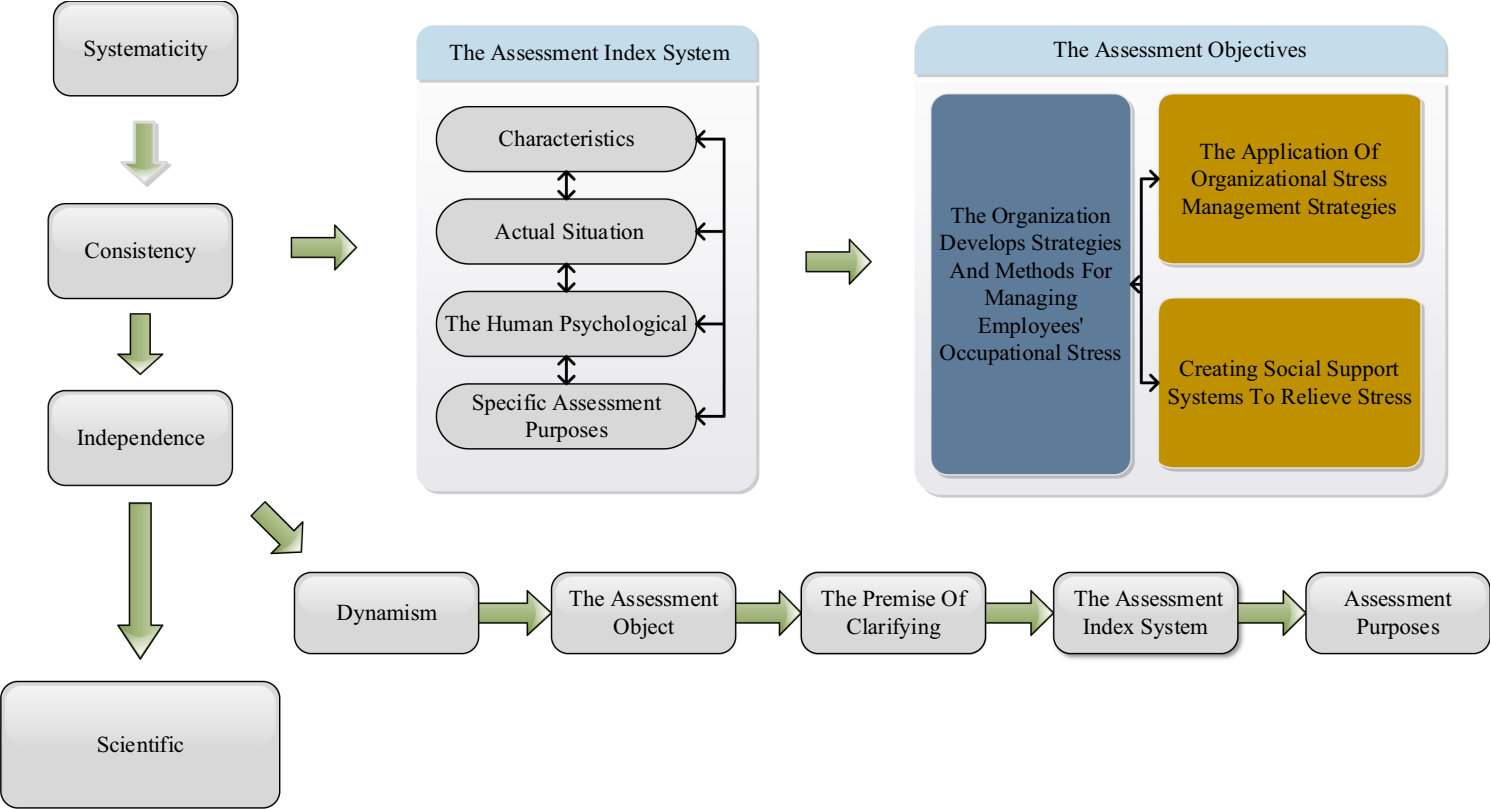
The process of employee psychological stress assessment.

This study complies with the principles of systematicity, consistency, independence, operability, science, and dynamism to ensure the assessment index system can meet the needs of the assessment objectives. It combines the company’s characteristics and actual situation to be fact-based and targeted. The construction of the human psychological assessment index system should first clarify assessment objects as objects based on specific assessment purposes. With different assessment objects and different assessment purposes, the construction of the assessment index system is not the same on the premise of clarifying the assessment object, setting clear objectives and goals, establishing the right direction, carrying out the work in an orderly manner, and finally building an assessment index system with practical value. Stress management is mainly defined as personal stress management and organizational stress management. Personal stress management is to identify, evaluate and actively deal with stress from individuals’ perspectives to relieve stress and improve the quality of life and work efficiency. Organizational stress management mainly refers to stress management measures implemented within an organization, i.e., the organization develops strategies and methods for managing employees’ occupational stress based on their physical and mental health and performance and directs them in a managerial manner, with a core focus on reducing the negative impact of stress and psychological burden on employees (Tambe et al., 2019). For the application of strategies for personal stress management, the current methods commonly used in society are mental and emotional management, self-direction of stress, time management, expansion of social support networks, etc. For the application of organizational stress management strategies, the following methods are commonly used in society: creating social support systems to relieve stress (including instrumental support, emotional support, cognitive support, etc.), job redesign to alleviate stress (including job rotation, job enlargement, job enrichment, etc.), personnel selection to relieve stress, employee participation activities to relieve stress, etc. The process of employee psychological stress assessment is shown in Figure 1.

At present, the company lacks the psychological assessment indexes for the grassroots employees, grassroots and middle managers at all levels, so this paper takes middle managers, grassroots managers, and grassroots employees as the assessment objects respectively, and establishes the staff assessment index system for staff recruitment, staff selection, and staff training to improve the company’s staff assessment system, improve the effectiveness of human resources and achieve corporate goals. Specific assessment objectives such as (1) The purpose of recruitment and selection: through the establishment of the assessment index system, you can set more goals and quantifiable and comprehensive assessment indicators to provide the basis for the company’s recruitment and selection of excellent, suitable, and outstanding employees, with the core competitiveness of the staff a critical factor in achieving the strategic goals of the Enterprise. (2) The purpose of selection and allocation: through the establishment of the assessment index system, set scientific and comprehensive, in line with the requirements of the position of the quantitative assessment indicators, so that the company in the internal staff to select the grassroots managers with management potential, strategic vision of the middle management, and constantly improve the effectiveness of the Enterprise to achieve sustainable development. (3) The purpose of training and development: through the establishment of targeted assessment indicators, to discover the knowledge, ability, and quality defects of employees, to discover the potential and shortcomings of managers, to focus on training issues, set personalized training needs, through different training methods, to improve the level of employee ability and knowledge, and constantly improve the management quality of managers, while creating core competitiveness for the Enterprise, to achieve the enterprise and employee win-win situation. (4) The purpose of human resource effectiveness: Through the establishment of a full range of employee assessment indicators, improve the employee assessment system, provide a scientific basis for enterprise recruitment, selection, and training, and continuously improve the effectiveness of human resources, enhance the core competitiveness of the Enterprise, and achieve the strategic goals of the Enterprise.

This chapter first introduces the introduced PLS (Partial Least Squares, PLS) structural equation model, including its establishment steps and advantages in this study. Structural equation modeling is a method for constructing, estimating, and testing causal models. The model contains both explicit variables that can be observed and latent variables that cannot be directly observed. Structural equation modeling can replace multiple regression, path analysis, factor analysis, and covariance analysis to analyze the effect of individual indicators on the total and the interrelationship among individual indicators. Secondly, based on the review of the theories related to employee’s mental health, occupational tasks, and personal resources, combined with the basic principles of the PLS structural equation model, the interrelationship between mental health and occupational schemes and emotional resources of frontline employees in the manufacturing industry is explored (Tanjung, 2020). Relevant hypotheses are proposed based on previous studies. PLS structural equation model is generally composed of two parts: external model and internal model, which can also be called measurement models and structural models. The measurement model usually reflects the relationship between latent and observed variables.

In contrast, the structural model describes the relationship between latent variables, that is, the causality of the model. a critical role of the PLS structural equation model is to estimate the values of latent variables, and each latent variable in the PLS structural equation model is a linear combination of its associated observed variables, which can well reflect the relationship between the latent variables and the corresponding observed variables The PLS structural equation model can be considered as a complete proxy for the observed variables. Structural equation modeling has become a standard tool in empirical analysis to test the relationship between observed and latent variables and between latent and latent variables. The PLS approach to study from a simple structural equation model of two variables:

Where *a*1 and *a*2 are the independent and dependent variables and are the same latent variables. *Lx* and *Ly* are the estimates of PLS, and *X*1–*X*4 and *Y*1–*Y*3 are the observed variables of *a*1 and *a*2, respectively; *β*12 denotes the correlation of latent variables *a*1 and *a*2. Model setting: Based on the path diagram, model Equations (1) and (2) are set:


(1) $$X_{h-1}=\underset{o=1}{\sum }\frac{\pi_{h-o}-\left(\pi_{h}-a1\right)}{\sqrt{\lambda_{h+1}+1}}$$

(2) $$X_{k-1}=\underset{o=1}{\sum }\frac{\pi_{k+o}-\sqrt{\left(\pi_{k}+a1\right)}}{\sqrt{\lambda_{k-1}-1}}$$

Where $\pi_{n}$ is the loading of a1, $\pi_{k}$ is the loading of *a*2, $\lambda_{h}$ , and $\lambda_{k}$ are the residuals, and $\pi_{ho}$ $\pi_{ko}$ are the intercepts. Also, the model needs to satisfy the relationship requirements of Equations (3) and (4):


(3) $$E\left(X_{h}+a1\right)=\sum \frac{\pi_{h}+a1}{\pi_{h+o}-\pi_{h}}+\left(\pi_{h}-a1\right)$$

(4) $$E\left(X_{k}-a2\right)=\sum \frac{\pi_{ko}}{\pi_{\left(k-o\right)}-\sqrt{\pi_{k}+a_{2}}}$$

Non-correlation needs to satisfy the requirements of Equation (5):


(5) $$\lambda_{h}-a1=\sum r\left(\lambda_{k}+a2\right)+r\frac{\lambda_{h}-\lambda_{k}}{\sqrt{\lambda_{k}-a}}$$

The model must satisfy Equations (6) and (7) requirements:


(6) $$a2=\sum \left(\beta_{0}-\beta_{1}\right)\times \sqrt{a1-e_{1}}$$

(7) $$E\left(a1-a2\right)=\sum \left(\beta_{0}+\beta_{1}\right)\times a1$$

A nonparametric test is used to evaluate the validation effect of the model. In the PLS structural equation model, since all loadings are standardized, the Average Variance Extracted (AVE) equals the joint degree. The greater the standard degree, the better the convergence effect; generally, the joint degree index should be greater than 0.5. *R*^2^ is used to evaluate the explanatory power of the structural model, and each internal Equation can be assessed using *R*^2^ to judge its explanatory power. The mean value of *R*^2^ of internal relations, the average explained variance, indicates the predictive ability of internal links, regardless of external relations. Generally, *R*^2^ should be greater than 0.45, redundancy is used to evaluate the overall predictive relationship of the model, and redundancy is the product of the joint degree and the squared multivariate correlation, i.e., *F* is the product of H2 and *R*^2^.

### Analysis of enterprise human resource loss

**Figure 2:**
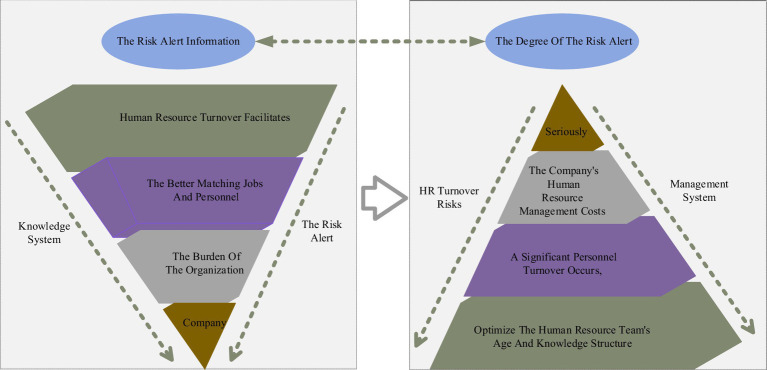
Framework of enterprise human resource turnover model.

Human resource turnover refers to high frequency and large-scale human resource movement with a turnover rate exceeding the industry-stopping norm. Overall, human resource turnover facilitates the better matching of jobs and personnel, leading to healthy business growth. It can reduce the burden of the organization when human resources resign due to their inability or health condition to do the job. Suppose the human resource turnover rate is kept within the normal range. In that case, it can encourage the organization to absorb new strengths and optimize the human resource team’s age and knowledge structure. Of course, if the human resource turnover rate exceeds the normal range, i.e., if a significant personnel turnover occurs, it will substantially increase the company’s human resource management costs, leading to a reduction in the quality of the company’s services and seriously affecting the stable development of the company (Tërstena et al., 2020). The function of the HR turnover management system is to prepare emergency countermeasures or management measures in advance in case of various risk causes and to call for appropriate measures according to the type and nature of the risk alert information and the degree of the risk alert once it is detected that the company is alerted to HR turnover risks. Companies recruiting talents overemphasize work experience, rarely consider whether the candidate’s values are unified with the company, and lack scientific talent selection procedures, making the company get the much-needed talents quickly. Still, in the long run, it increases the turnover rate of the company’s employees. The measures in the HR turnover risk management system are primarily thoughtful and suggestive so that decision-makers will not be overwhelmed and make mistakes when a risk alert is issued and will guide the relevant departments that issued the risk alert to seek more specific implementation plans according to the prompts of the pre-control measures. The framework of the enterprise human resources turnover model is shown in Figure 2.

The scientific approach of the whole HR turnover risk management system can be operated according to the following process: the internal and external information related to HR turnover risk is entered into the risk management system, stored, processed, screened, and inferred, and then separately entered into the risk indicator system, and after each indicator is measured, a comprehensive evaluation result is obtained through the PLS structural equation model to decide whether to issue a risk alert or not. Then, based on the evaluation results, the management measures in the risk management system are invoked. When the decision-makers receive the risk alert signal, they must make general inferences based on the risk alert signal in a short period to reveal what the risk alert signal means, search for the causes of the risk, and apply the tips of risk management measures (but not limited to such tips of management measures) to study the concrete countermeasures and standards, and implement them as soon as possible Implement them as quickly as possible. In the implementation process, implementation’s effectiveness If the results are unsatisfactory, the causes must be identified, corrected, and remedied promptly. The risk alert information base must be updated promptly, with new information added, and old news has proven to be incorrectly deleted for future reference and use.

**Figure 3:**
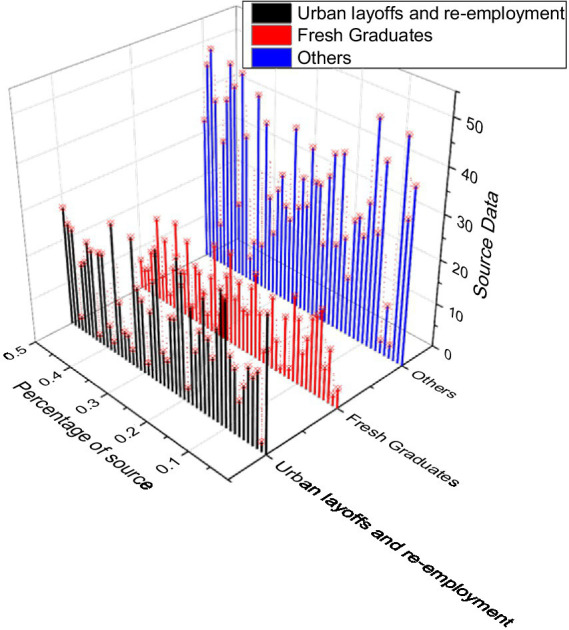
Comparison of human resources sources.

Analyzed from the source of employment perspective, most of Enterprise’s human resources are obtained through graduate recruitment. The proportion of laid-off human resources employed had increased, from which we can easily see that it can help the city solve part of the problems related to the employment of the new population. However, the relatively low proportion of employees and migrant workers proves that there is always a part of the problem that has not been fundamentally solved in the human resources sources used by Rujia Hotels. In addition to the abovementioned issues, some problems have strong common characteristics. Namely, the recruitment channels are too narrow, and the development process only uses the Internet and newspapers to conduct human resources recruitment activities. This does not allow the practical utility of training colleges and institutions to be fully utilized. The comparative chart of human resources sources is shown in Figure 3.

Human resource management should enable the company to achieve its strategic goals and employees to realize their career planning as an essential link in the enterprise development process. Human resource management should be done from both Enterprise and employee perspectives. Based on the original system of human resource management of the company, the existing enterprise resources are effectively integrated, re-optimization research is conducted, and human resource management optimization programs suitable for the development of the company are proposed so that the human resource needs of various departments, mainly production and sales departments, can be met. The Enterprise’s overall productivity can be improved to drive the Enterprise to achieve rapid transformation and implement the strategic goals of economic development in the new era. (1) Improve the human resource management environment: An excellent human resource management environment directly affects the effectiveness of the Enterprise, which in turn involves the salary and benefits of each employee. Therefore, employees to business managers should enhance their understanding of human resource management, fully understand the importance of human resource management to the development of enterprises, and establish a scientific human resource management concept. (2) Retaining employees: With the business environment proposed, enterprises gradually recognize the importance of employees; if enterprises want to survive and develop, they need to retain employees. Companies in the selection of employees at the same time, employees are also measuring the Enterprise, in consideration of the salary package will also consider the future development of the Enterprise, the enterprise platform is suitable for their better development. The company wants to retain employees with a high salary package, a sound human resource management system, staff training, and development; each link cannot be missing. (3) Improve the competitiveness of the company: Human resource management contains staff recruitment, training, performance management, interpersonal management, and other links. The company optimizes human resource management to guarantee the regular operation of the Enterprise while creating more value for the Enterprise, comprehensively improving the comprehensive strength of the Enterprise, and being comfortable in the face of market competition.

## Analysis of results

### Analysis of the model for measuring the psychological stress situation of enterprise employees

As mentioned in the introduction about the PLS structural equation modeling method, compared with other structural equation modeling methods, the PLS method does not require that the data used for the analysis conform to a normal distribution. Still, it does require that the variables tested have unidimensionality. To determine the suitability of the PLS structural equation modeling method chosen for this study, the normality and unidimensionality of the data were first examined before the data were analyzed (Androniceanu et al., 2020). The results of three variables of mental health, occupational tasks, and personal resources, and the factors they contained, were significantly different from the original hypothesis that the sample data followed a normal distribution and could be considered not to follow a normal distribution at least at the 0.003 level of significance. The KMO value of EFA was 0.916, and the significance level of Bartlett’s spherical test was less than 0.001. According to the KMO test criteria, the variables were correlated with apparent structure, suitable for factor analysis, and three principal components were extracted from the results. The overall variance explained 63.681%, each item clustered on the corresponding main element and had the maximum loading value on that central component. All of them were greater than 0.5, and the loading values on other extracted features were much smaller than the top loading value; the scale had good structural validity. SPSS is powerful, and SPSS has complete functions of data input, editing, statistical analysis, report, graph production, etc. It provides an entire data analysis process and covers a full range of statistical analysis methods for data, such as exploratory analysis of data, partial correlation, analysis of variance, a nonparametric test, multiple regression, logistic regression, etc.

**Figure 4:**
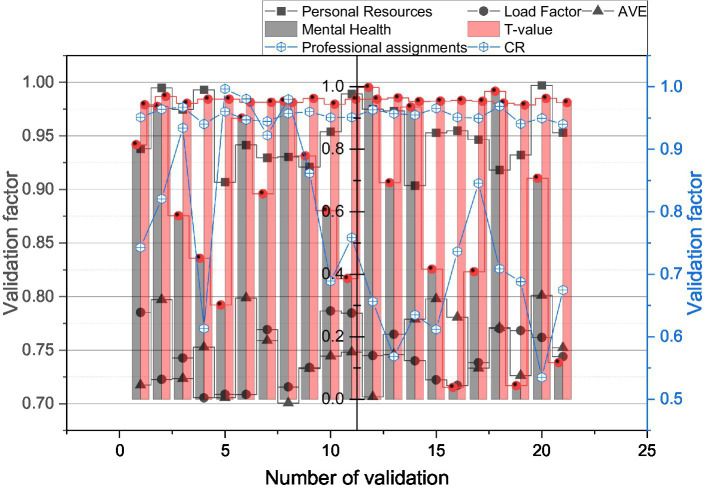
Results of validation factor analysis.

Meanwhile, SPSS can read and output files in various formats, such as reading files generated by dBASE, FoxBASE, and FoxPro, and also converting graph and table files directly to Word, Excel, PowerPoint, TXT, HTML, PDF, and other formats for saving. The validated factor analysis was then conducted on the 21 factors of these three variables using Smart PLS 3.2.6. The results are shown in Figure 4, with the values in bold in the table indicating that the values are the square root of AVE. The results loading coefficients of the factors ranged from 0.675 to 0.899, the *t*-values ranged from 12.608 to 25.651, and the *t*-test values of the loading coefficients were all significant, with CR ranging from 0.896 to 0.976 and AVE ranging from 0.805 to 0.872, indicating good convergent validity of the scale.

**Figure 5:**
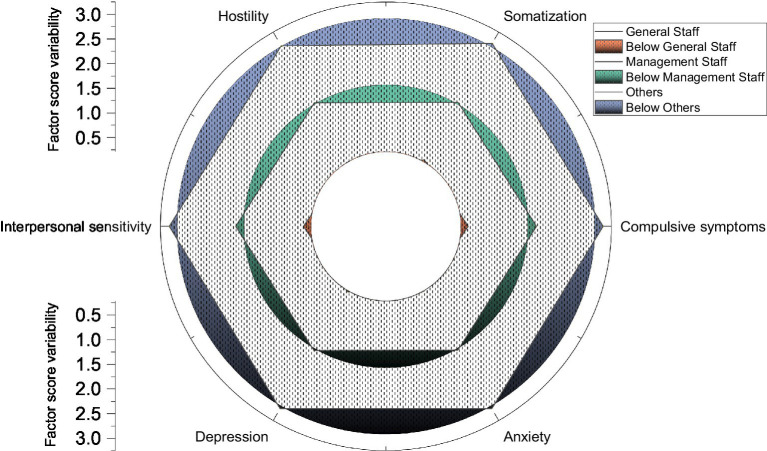
Comparison of the total mean score and the variability of each factor score of the symptom self-assessment scale for employees in different departments.

The study examined the differences in the company’s employees using different employees as grouping variables. The management employees scored higher than the general employees on the total mean score and each factor of the symptom self-assessment scale, which indicated that the public employees had a significantly lower overall mental health level than the management employees. A comparison of the differences in the total mean scores and the factor scores of the symptom self-assessment scales of employees in different departments is shown in Figure 5. From the difference test, there is a significant difference in the mental health level of employees in various work departments, and the overall mental health level of general employees is lower than that of management employees. Further statistics were done on the detection rates of employees in other departments on each factor, and it was found that the detection rates of obsessive–compulsive symptoms were high. The main problem, in general, was obsessive–compulsive symptoms (37.70%), followed by somatization (28.14%) and hostility (26.78%), and the main problems of management employees were obsessive–compulsive symptoms (41.26%), interpersonal sensitivity (26.21%), depression (22.82%), and anxiety (22.82%).

From the assessment results and data, all factors of the middle managers who participated in the assessment are higher than the standard model, which indicates the current positive detection rate of mental health and the detection rate of psychological problems of the company’s middle managers. The overall level of mental health is low, lower than the general level of the average national population. The dynamic employee turnover management system includes two major sections: early warning analysis of employee turnover and special countermeasures. The countermeasures mainly include the system preparation, crisis response measures, and the employee turnover management system’s evaluation system. The employee turnover early warning model establishes the information monitoring mechanism, and the employee turnover tendency assessment cycle is synchronized with the performance assessment. When the employee turnover tendency is high, the company will send a suitable candidate to conduct in-depth and repeated communication with the employee to understand the employee’s conditions for not leaving and the retention conditions prescribed by the senior assessment to retain the employee (Baharun et al., 2021). The employee will continue to participate in the next phase of the evaluation after successful retention, and the employee will need to evaluate the loss of the employee after unsuccessful retention, arrange the replacement work for the position in time, and require the employee to entirely hand over the work and sign the technical confidentiality agreement before leaving. The new employee turnover management system can effectively advance the company’s employee turnover management and achieve the effect of pre-emptive employee turnover control.

### Employees psychological stress situation assessment test for companies to reduce human resource turnover

**Figure 6:**
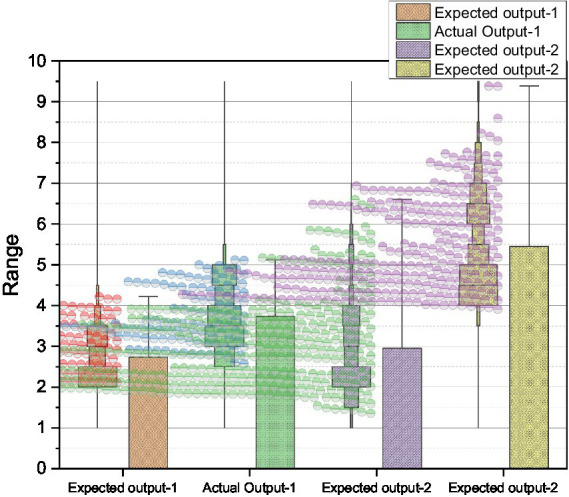
Comparison of the actual and expected output of training and test samples.

The function of the HR turnover risk management system is to prepare emergency countermeasures or management measures in advance for various risk causes and to call for corresponding measures according to the type and nature of the risk alert information and the degree of the risk alert once it is found that the company has issued an HR turnover risk alert. The HR turnover risk management system measures are primarily thoughtful and suggestive so that decision-makers will not be overwhelmed when a risk alert is issued and will be guided to seek more specific implementation plans per the prompts of the risk control measures. We will focus on companies’ HR turnover risk management measures in the following. The following process can follow the scientific approach to the entire HR turnover risk management system: internal and external information related to HR turnover risk is entered into the risk management system, stored, processed, screened, and inferred, and then separately entered into the risk indicator system, and after each indicator is measured, a comprehensive evaluation result is obtained through the PLS structural equation model to decide whether to issue a risk alert. Then, based on the evaluation results, the management measures in the risk management system are invoked. When the decision-makers receive the risk alert signal, they must make general inferences based on the risk alert signal in a short period to reveal what the risk alert signal means, find the cause of the risk, apply risk management measures to prompt the study of specific countermeasures and standards, and implement them as soon as possible. In the process of implementation, the effectiveness of implementation must always be evaluated. If the results are unsatisfactory, the causes must be identified and promptly corrected, and remedied. The risk alert information base must be updated promptly to add new information and delete old, proven wrong information for future reference and use. The actual and expected outputs of the training and test samples are compared, as shown in Figure 6.

**Figure 7:**
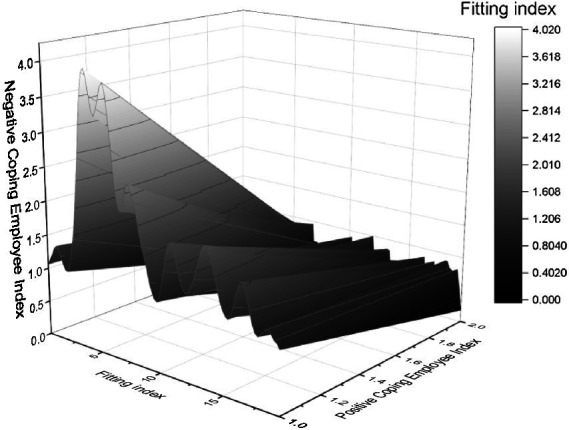
Modified model and fit index.

The comparison of the actual output and the expected output of the risk signal curve can be seen, and we can see that the error between the two is small, so a PLS structural equation model on the risk of corporate human resources turnover has been learned and trained successfully. Of course, the model is only adapted here for the convenience of the research problem; in establishing the PLS structural equation network’s comprehensive evaluation model, all possible risk indicators should be obtained for risk evaluation according to the basic requirements. There are significant differences in employees’ psychological capital and the elements of psychological capital in different departments. Overall, the psychological capital of general corporate employees is lower than that of management employees. Further post the significant difference in the self-efficacy elements of psychological capital level between available corporate employees and management employees, indicating that the same level of confidence exists in the face of work for employees in both departments. Significant differences exist in the dimensions of hope, optimism, and resilience, and employees in other departments have higher levels in all three sizes than the general employees. AMOS 19.0 software can quickly help users analyze regressions, factors, and other correlations as an extension of multivariate analysis methods such as ANOVA, providing rich support for users’ theories. The software’s analytical approach allows users to produce statistics on relevant data. The study used AMOS 19.0 path analysis to explore the predictive effects of employee burnout, coping styles, and psychological capital on psychological wellbeing (note: both positive and negative correlations presented in this model indicate only the mean correlation of each scale). The results of the model test of the chi-squared degrees of freedom ratio were 1.589 < 3.000, GFI value = 0.989 > 0.900/CFI = 0.999 > 0.900, NFI = 0.987 > 0.900, IFI = 0.997 > 0.900, RFI = 0.964 > 0.900, RMSEA = 0.038 < 0.080, all of which reached the model fit criteria model is better, the model after the correction of and the fit index are shown in Figure 7.

The study of psychological stress assessment found that employees’ coping styles affect their psychological health, psychological capital, and burnout level. Employees who adopt negative coping techniques have higher levels of burnout, which leads to a decrease in their psychological health. In comparison, employees who adopt positive coping styles have lower levels of burnout, which in turn promotes the improvement of their psychological capital and psychological health. Thus, we found that the level of burnout and the coping style of employees play a mediating role in the influence of psychological health. The case of burnout can effectively reduce the occurrence of psychological problems and improve occupational mental health by enhancing the psychological level of metro employees. It does not necessarily lead to changes in employees’ emotions and behavior. What plays a key role is the interpretation made by the employee of the psychological contract change, breach, or violation. The two main factors that influence the employee’s interpretation process are the attribution of the change, breaking, or violation of the psychological contract and the perceived fairness of the employee in such a dynamic process of the psychological contract. Attribution of events is an essential factor influencing employee behavior and emotions after a psychological contract break. Suppose employees attribute a deliberate breach of the contract to the company. In that case, the ensuing reaction will be harmful, and employees will condemn the actions of the company’s managers and reduce their performance or even leave the company. If it is attributed to a difference in understanding, the reaction is much better. Therefore, this point is an opportunity for managers to make a difference. Both management scientists and psychologists have discussed the issue of fairness, an essential principle of organizational management. Research has found a complex interaction between attribution and perceptions of fairness: if employees attribute a different understanding of the psychological contract, the role of justice seems to be absent; and if the attribution is because the organization deliberately violates the contract, the part of fairness is essential; and conversely if employees feel strongly unfairly treated, this can seriously affect their attribution conclusions and can amplify the psychological contract being violated. This can create a negative feedback loop and even harm the organization. In management practice, specific objective reasons are likely to produce the destruction of the psychological contract of employees; at this time, if the management is indifferent to the treatment of employees will be a psychological contract violation, back to the failure of the Enterprise to achieve the original promise, on the contrary, if the enterprise managers to give employees care, he will be at this time attributed to objective rather than corporate. Therefore, the appropriate use of management skills and the psychological contract will be broken to give employees a reasonable explanation is very beneficial.

## Conclusion

With the current economic conditions and environmental changes, enterprises are bound to face a new round of enormous social and economic development challenges. At the same time, as the level and degree of economic openness increases, the market competition that enterprises are bound to face is also rapidly intensifying, and talent has gradually become the key for enterprises to determine their competitive advantage and grasp future development opportunities. Implementing human resource management scientifically, rationally, and efficiently has become an urgent need in the current economic globalization. Guided by the concept of modern enterprise human resource management, enterprises have systematically and comprehensively studied their organizational structure, talent structure, development, and management strategy, management system construction process, and the main problems in human resource management and redesigned their human resource management system from a general perspective, thus completing This fundamental change in the human resource management model of the Enterprise and the formation of a human resource management system that matches the long-term development strategy of the Enterprise can genuinely solve a series of problems faced by the Enterprise in human resource management. This paper analyzes and improves the main reasons for the risk of human resource turnover in enterprises; establishes a system of indicators for the risk of human resource turnover in enterprises, explains the content of each last-level hand and its measurement method; a PLS structural equation evaluation model for the risk of human resource turnover in enterprises, and gives an application example; proposes countermeasures to reduce the risk for each main reason of the risk. The analysis of burnout, coping style, and psychological capital has a predictive effect on assessing employees’ psychological stress status. Employees’ burnout and coping style impact their psychological health level, and the evaluation of psychological stress status plays a significant mediating role.

## Data availability statement

The original contributions presented in the study are included in the article/supplementary material, further inquiries can be directed to the corresponding authors.

## Ethics statement

Ethical review and approval was not required for the study on human participants in accordance with the local legislation and institutional requirements. The patients/participants provided their written informed consent to participate in this study.

## Author contributions

CL and WM participated in the related research work in this paper. CL was organized and completed this manuscript. They agreed to publish. All authors contributed to the article and approved the submitted version.

## Conflict of interest

The authors declare that the research was conducted in the absence of any commercial or financial relationships that could be construed as a potential conflict of interest.

## Publisher’s note

All claims expressed in this article are solely those of the authors and do not necessarily represent those of their affiliated organizations, or those of the publisher, the editors and the reviewers. Any product that may be evaluated in this article, or claim that may be made by its manufacturer, is not guaranteed or endorsed by the publisher.
